# Innovation in Rural Health Services Requires Local Actors and Local Action

**DOI:** 10.3389/phrs.2022.1604921

**Published:** 2022-09-14

**Authors:** Dean Carson, Robyn Preston, Anna-Karin Hurtig

**Affiliations:** ^1^ Department of Epidemiology and Global Health, Umeå University, Umeå, Sweden; ^2^ School of Health, Medical and Applied Sciences, Central Queensland University, Rockhampton, QLD, Australia

**Keywords:** community participation, innovation, rural health services, local actors, local action

## Abstract

**Objectives:** We examine the role of “local actors” and “local action” (LALA) in health service innovation in high-resource small rural settings and aim to inform debates about the extent to which communities can be empowered to drive change in service design and delivery.

**Methods:** Using an adapted roles and activities framework we analyzed 32 studies of innovation projects in public health, clinical interventions, and service models.

**Results:** Rural communities can investigate, lead, own and sustain innovation projects. However, there is a paucity of research reflecting limited reporting capacity and/or understanding of LALA. Highlighting this lack of evidence strengthens the need for study designs that enable an analysis of LALA.

**Conclusion:** Innovation and community participation in health services are pressing issues in small rural settings where population size and distance from health infrastructure make service delivery challenging. Current reviews of community participation in rural health give little insight into the process of innovation nor understanding of how local actors produce improvements in innovation. This review outlines how communities and institutions can harness the essential role of LALA in supporting health innovations.

## Introduction

This paper examines the role of “local actors” and “local action” (LALA) in health service innovation in small rural settings. It is intended to inform debates about the extent to which small rural communities can be empowered to drive change in service design and delivery. The review targets case examples of innovation in a search for evidence that LALA impacts not only how innovation occurs, but the effectiveness of its outcomes.

There is widespread agreement about the need for community participation in rural health service design. While there is little clarity about what is meant by community participation, it is typically depicted as a process whereby “they” who represent “the community” serve to inform or influence “we/they” who operate health services [1] Three reviews of research into community participation [1–3] in rural health have revealed some evidence of impact on health outcomes, but limited evidence of impact on intermediate processes like service design. In contrast, three reviews of “innovation” in rural health [4–6] have suggested the importance of community participation in design processes but offer little evidence of health impacts.

It has been argued that both the need for innovation and the need for community participation are more pressing in small rural settings where population size and distance from larger service centers make it “impractical to deliver the same services as are enjoyed in urban areas.” [4]^p258^ Precise definitions of “small rural” are elusive [7], but Hancock et al. (2001) [8] and more recently Scott et al. (2013) [9] have suggested that populations smaller than 5000 or 6000 residents which are not readily accessible catchments of larger centers are unlikely to be able to support “standard” models of health services. “Rural” geography models in Australia [10], Sweden [11] Canada [12] and US [13] variously suggest towns or catchment areas with between 5000 and 15,000 residents as something between “urban” and “remote” areas. These definitions also include specification of a minimum distance to a larger center, which may be a “hard” definition as with the Australian model (10 or 15 or 20 km depending on the size of the larger center) or a “soft” definition as in Canada (“commuting distance”). It is important to note that small rural does not include the “remote,” which is characterized by extreme distance from service centers and vastly different processes of governance [14].

In these contexts, the dividing line between “community” and “service providers” is blurry if not absent when those service providers also live locally [15]. Consequently, rather than referring to “community participation” dichotomously, this paper is interested in what we term “local actors” and “local action” (LALA). Local actors can include locally resident health service providers as well as other community members. “Local action” incorporates the roles that local actors play in the innovation process.

The term “innovation” in rural health also defies precise definition, although Wakerman (2009) [6]^p21^ borrowed from Greenhalgh et al. (2005) [16] definition as being a “novel set of behaviors, routines and ways of working that are directed at improving health outcomes, administrative efficiency, cost effectiveness, or user’s experience and that are implemented by planned and coordinated actions.” Our research is more interested in a process-perspective, drawing on innovation science to examine not so much the “behaviors and routines” that map to a novel service approach, but the processes that lead to the emergence of new ways of doing things. These processes can be depicted as a series of (non-linear) activities which are described below [17].

In conducting this review, we are aware that our results will be influenced by the nature of academic research. Academic literature is likely to focus on those parts of the innovation process which relate to the research and development activities of academic actors. However, we also expect that academic literature should be highly sensitive to LALA given the ethical parameters which frame academic research and the “community engaged” and “socially responsible” paradigms seen as driving rural health education and scholarship. Nevertheless, in paraphrasing Preston et al. (2010) [2], we are aware that the absence of evidence for LALA in rural health innovation does not mean an absence of LALA in practice.

### Community Participation in Rural Health Innovation

Reviews of community participation in in rural health suggest a hierarchy of participation types. This does not include what Kenny et al. (2013) [1] term “non-participation,” which involves being serviced or manipulated as users or research subjects with no real influence on what is done or how it is done [3]. Actual community participation sits on a continuum ranging from consultation (“tokenism” according to Kenny et al.) [1] to ownership (“power”). Tokenism includes informing, consulting and “placation”, while power includes partnership, delegated power, and ownership.

Preston et al. (2010) suggest that limited research at that time had focused on the contributions of community participation to health service design and health outcomes [2]. Bath and Wakerman (2015) asserted that evidence for the impacts of community participation were stronger in communities which were more marginalized and distanced from “mainstream” primary care systems [3]. They used the example of Indigenous communities, where the need for community-specific service models has long been recognized. Perhaps the evidence for impact of community participation is stronger in these settings because participation is essentially mandated, there are more or less formal structures of leadership and community representation, and research into service models in those communities consequently is more likely to focus on community participation.

More broadly, however, Kenny et al (2013) note the difficulty in continuing to find new leaders for community participation initiatives, reflecting persistent challenges in smaller rural communities of volunteer fatigue and systemic exclusion of community members who sit outside the dominant social structures [1]. There was also a tension between formal structures of community representation (such as local government) and structures that might be preferred for rural health initiatives. In small rural settings, the “local” government may not be local at all (headquartered elsewhere) or may not represent a broad range of community interests.

Our review extends the concept of community participation beyond simply “communal” models (where people are brought together to collectively represent the community), and we reject the dichotomy between “community” and “health service providers” in cases where those providers are also members of the community [18]. We define LALA as:

A local actor is any individual or group who lives in the location where the innovation is taking place.

A local action is any non-passive role played by a local actor in the innovation process.

Wakerman (2009) [6] and Wakerman and Humphreys (2011) [5] focused their reviews of rural health innovation on primary care and referenced both “community readiness” and “public participation” in their proposed models of enablers of rural health innovation, but these were not discussed in the text, and the process by which the models emerged from the research is unclear.

Wakerman and Humphreys (2011) [5] describe four types of service innovations—discrete (relating to a specific primary care task), integrated (involving multiple tasks or services), comprehensive (typically across a population sub-group) and outreach (providing services at a distance). They claimed that discrete and integrated models are more appropriate for larger communities, while comprehensive and outreach models are more appropriate for smaller communities. Asthana and Halliday’s (2004) [4] review encompassed more than primary care, but the typology of innovations they proposed was similar, with various forms of integrated care and outreach. They also suggested that smaller rural contexts would benefit from novel approaches to “intermediate care” to facilitate transitions to and from (distant) specialist services, and substitution of one type of professional with another (such as nurse practitioners, or physician-pharmacists).

Beyond service models, innovation may include public health interventions, clinical interventions, uses of eHealth, information distribution to build health literacy, and patient or user engagement [4]. Recent attention has largely focused on eHealth [19] where there has been some discussion about the extent to which eHealth local actors can influence tech companies and central health departments.

The rural health innovation reviews support the common idea that rural health and care systems need to do more with less—“innovation on a shoestring” according to Mathieu et al. (2020) [20]. While small budgets may be viewed as a hinderance to rural health innovation, the approaches to innovation that they dictate—small steps and “optionality”—have been promoted as a more robust approach than large projects which carry higher risk of failure or “lock-in” to unsuitable models [21]. “Optionality,” in particular, may be important as it allows lessons learned or resources gathered for one purpose to be used in other ways (Petrie et al. [21] give the example of teleconferencing facilities being used for non-health purposes). Similarly, Hodge et al (2016) [22], postulated that distance from the agencies which typically drive health innovation (provincial health departments, universities) can both limit local influence and provide opportunities for increased local action as local actors “fly under the radar.”

### Innovation Roles and Phases: A Framework

There is abundant literature describing both roles and activities associated with innovation [17]. Thune and Mina (2016) describe four broad roles in health service innovation processes: idea generation; idea development; selection; and implementation and dissemination [23]. Our synthesis of these and other “roles” frameworks [24] (see horizontal axis in Table 1, with definitions below) leads to explication of five roles which could be assumed by local actors.

**TABLE 1:** Local actors for innovation processes in Health Services: Roles and activities framework (Sweden, Australia. 2022).

Roles →	Stimulator- initiator	Facilitator	Researcher- developer	Leader-owner	Translator- maintainer
Phases↓					
Exploration	Central to the process of idea generation and involving proactive engagement in activities such as writing or calling for project proposals, or establishing project teams	A role extending throughout the innovation process, and involving lobbying, mediating between stakeholders, recruiting participants or lending credibility to the project	Central to the ongoing tasks of refining ideas, selecting from alternative proposals, conducting experiments, creating tools or guidelines, clinical testing and evaluation	In this context we are referring to leadership or ownership of the process up to the point of commercialization. This is the role which has responsibility for the conduct of the innovation process—which has ultimate responsibility for what is done during the process and how it is done	In this context we refer to the roles involved in translating the project into ongoing practice, which might include educating users, establishing longer term governance structures, securing ongoing funding, and ensuring that sufficient policies and procedures are in place to support implementation
Preparation					
Implementation					
Sustainment					

Moullin et al. (2019) have promote the *Exploration, Preparation, Implementation, Sustainment (EPIS)* framework for understanding implementation of evidence-based practices in health and medicine (vertical axis, Table 1) [25].

Aarons et al. (2011) description of the EPIS framework defines the exploration phase as developing “awareness of either an issue that needs attention or of an improved approach to an organizational challenge” [26]^p6^. The preparation phase involves the research, policy making, and consultation which leads to the decision to test (or adopt directly) a particular innovation idea or set of ideas. Implementation is the active task of putting the idea into practice through experimentation, physical construction, producing or procuring software or technology, formally adopting new guidelines and so on. Sustainment encompasses the securing of resources, establishing policies and protocols, and dissemination (through education and training or knowledge mobilization) that allows the initiative to become “standard practice.” It is often the case that academic attention to the innovation process “departs” at the point of implementation, but our review at least aims to allow for the possibility of investigation of sustainment of an initiative either in concept (plans for sustainability) or in practice (longer term evaluation).

The phases of the innovation process are not necessarily linear, and certainly overlap temporally and conceptually (for example, research and testing can continue throughout and beyond the implementation phase). All the roles (horizontal axis of Table 1) can be undertaken during all the phases in the EPIS framework. As with Thune and Mina’s (2016) review of hospitals as innovators, our framework suggests that local actors CAN engage in all roles and in all phases of the innovation process, but there has not yet been a synthesis of empirical evidence of the extent to which they do so engage, and the impacts of engagement on the outcomes of health service innovation in small rural contexts [23].

## Methods

The review is informed by six questions:

What type of initiatives are considered innovations in these settings?

How do theories of innovation used in small rural health research consider the role and value of LALA?

Who might serve as local actors?

What roles do local actors play in innovation processes?

What innovation process phases can be impacted by local actors?

What is the evidence that LALA impact the quality of innovation processes and outcomes?

Ideally, questions 4 and 5 would be answered as a tabulation per Table 1 (role/phase), but an initial scan of the literature revealed an absence of the level of detail required for such analysis. Table 1 therefore stands as a conceptual model, with the analysis of results separated into the two dimensions of role and phase.

The research followed a structured review process [27]. The purpose of the review was to examine the roles of local actors and local action in health service innovation in small rural settings in high resource countries. *EbscoHost* was the primary database used for the review, with other databases (*PubMed, Web of Science*) providing no additional items. *Google Scholar* was used to access referenced and citing articles of interest not listed in *EbscoHost,* but none of these were included in the final set of papers. There was no time limit applied to the search, but only English language articles were included.

We are aware that there are many innovative models arising from LALA in middle- and lower-income countries. However, as we want initially wanted to influence LALA in our countries (Sweden and Australia) we started with settings that have similar resources and policy frameworks in common that comparably affect local action. It would be very interesting and useful to expand the search for all countries, which is a next step in our research.

Supplementary File S1 review process (Sweden. 2022) summarizes the review process and the inclusion/exclusion criteria. The review included only empirical studies (not reviews or commentary or editorial), peer reviewed full papers (not conference abstracts, for example), studies about human health, studies in High-income countries as defined by the World Bank [28] and studies pertaining to “rural” (and ultimately “small rural”) settings. This latter was defined as rural service catchments with populations of fewer than 20,000 residents and being more than 1 hour drive (or 50 km) from a larger center while being commutable within a day. Where this information was not provided in the paper, population data were drawn from the statistical bureau of the relevant country. The criteria for “small rural” was applied quite loosely, with the intention to err on the side of including both larger and more remote settings rather than risk excluding settings with small rural characteristics. Articles featuring multiple sites where at least one site was “small rural” were included. Articles (from the full-text screening phase) which did not identify local actors were excluded, and later (eligibility phase) articles which did not identify an active role for local actors were excluded. Screening and selection were done manually, given the nuance involved in identifying “small rural” and LALA.

A number of combinations of search terms were tested, mostly involving variations on the term “innovation” (“discovery,” “novel/new initiative/model/process/service,” “reform”), however ultimately adding additional terms for innovation did not affect the number of records initially identified. The final search phrase was simply “rural health innovation” and was applied to the full text of articles. The initial search returned 5687 articles, from which 1009 duplicates were removed. The first screening (title, abstract and keywords) excluded 4349 records, mostly because they related to low resource and non-rural settings. 329 articles were read in full, and 43 of these proceeded to the final screening (eligibility) phase. A further 11 were excluded at this stage because they did not involve a health initiative, or they did not identify an active role for local actors. Decisions to exclude articles at this point were made jointly by two of the three researchers.

## Results

The final review included 32 papers. Exactly half of these were case studies in the United States, and a further eight were from Canada. Australia (3) and New Zealand (2) were also represented on multiple occasions, while there was one article each from Norway, the United Kingdom, and Japan. Supplementary File S2 (Sweden, Australia. 2022) provides a summary of included papers (small rural context, intervention and local role).

### Summary

Results are summarised in Tables 2–5 which partition the 32 included papers according to “local actors” “levels of action” [1] which we have classified within the ownership-consultation continuum as being “completely local” (7 papers), “local initiated” (12 papers), “local active” (7 papers), and “local passive” (6 papers). Completely local papers (Table 2) had local actors as the only participants and through all phases. These seven papers were also the only ones where local actors served as evaluators. In Tables 2–5 we have added “a” to each of the 15 health professionals’ papers to indicate whether there was reference to any other level of community participation.

**TABLE 2:** Completely local cases (Sweden, Australia. 2022).

Paper	Location	Type of innovation	Type of local actors
[31]	Newfoundland, Canada	Governance/service model	Health sector research and development unit
[57]	Ontario, Canada	Clinical intervention	Informal community group
[47]	Arizona, United States	Prevention/public health	Health professionals^a^
[29]	Washington State, United States	Prevention/public health	Local government
[59]	Hokkaido, Japan	Governance/service model	Informal community group
[36]	Kentucky, United States	Prevention/public health	Health professionals
[44]	Tennessee, Louisiana, New Mexico, United States	Governance/service model	Health professionals^a^

a Reference to other local participants.

Local initiated papers (Table 3) covered projects that emerged locally, but had some external actors invited to participate in specific phases or perform specific roles.

**TABLE 3:** Local initiated cases (Sweden, Australia. 2022).

Paper	Location	Type of innovation	Type of local actors
[43]	Saskatchewan, Canada	Clinical intervention	Health professionals^a^
[38]	Missouri, United States	Knowledge/capacity building	Health professionals^a^
[34]	Wisconsin, United States	Prevention/public health	Formal community group
[39]	Cornwall, United Kingdom	Prevention/public health	Formal community group
[53]	Waikato, New Zealand	Governance/service model	Formal community group
[33]	Minnesota, United States	User/patient engagement	Health professionals
[36]	New Brunswick, Canada	Knowledge/capacity building	Health professionals^a^
[55]	New South Wales, Australia	Prevention/public health	Informal community group
[56]	Waikato, New Zealand	Prevention/public health	Formal community group
[30]	Ontario, Canada	Clinical intervention	Health professionals^a^
[50]	Finnmark, Norway	eHealth/technology	Informal community group
[42]	North Carolina, United States	Governance/service model	Health professionals

a Reference to other local participants.

Local active cases (Table 4) were typically initiated by external actors, but local actors were invited or appointed to active leadership and developer roles.

**TABLE 4:** Local active cases (Sweden, Australia. 2022).

Paper	Location	Type of innovation	Type of local actors
[44]	Western Australia	Clinical intervention	Health professionals^a^
[59]	Ontario, Canada	Clinical intervention	Informal community group
[49]	Illinois, United States	Clinical intervention	Health sector research and development unit
[32]	Queensland, Australia	Knowledge/capacity building	Informal community group
[60]	Arizona, United States	eHealth/technology	Formal community group
[41]	Texas, United States	eHealth/technology	Health professionals^a^
[48]	Indiana, United States	eHealth/technology	Health professionals

a Reference to other local participants.

Local passive cases (Table 5) had less active local actor participation but did include at least some advisory functions which were acknowledged as influencing the course of the project.

**TABLE 5:** Local passive cases (Sweden, Australia. 2022).

Paper	Location	Type of innovation	Type of local actors
[52]	Hawaii, United States	Clinical intervention	Formal community group
[45]	Iowa, United States	Governance/service model	Health professionals
[54]	Alberta, Canada	Prevention/public health	Informal community group
[51]	British Columbia, Canada	Governance/service model	Informal community group
[40]	California, United States	Prevention/public health	Health professionals
[37]	Alaska, United States	Prevention/public health	Health professionals^a^

a Reference to other local participants.

The review included ten articles relating to public health interventions (for example an examination of a model for preventing chronic disease [29]), seven articles relating to clinical interventions (such as a palliative care model [30]) and seven relating to service models (e.g., an emergency care model [31]). There were also four articles relating to eHealth initiatives, three to knowledge or capacity building (such as an investigation of the establishment of a partnership for research in Indigenous communities in Australia [32]) and an initiative to improve patient journey management [33].

The tables do not include the theories of innovation used in the literature, as only seven papers explicitly cited any theoretical framework at all. These focused on approaches to engagement or participation of local actors (university-community engagement models [34], “principles for community governence” [35]^p614^ and community based or socially accountable practice [35, 36]). One study used a decolonisation framework to direct their research on the impact of an Indigenous youth suicide prevention program in Alaska [37].

Four of the seven innovation-related theoretical frameworks were concerned with change management (theory of change [38, 39], “Kotter’s model of change” [41]^p2^ and an improvements model [40]. Another study employed a diffusion of innovation theory in evaluating a telepsychology initiative [41], and one investigated the long-term outcomes of four primary care service models in the US using a “6 domains of sustainability” framework [44]^p1613^.

In fifteen cases, the “local actors” identified were health service actors, and the nine made reference to other local participants are marked with an asterisk in the tables. Five of these involved an individual health professional [three nurses [33, 35, 36], pharmacist [52] and a clinical champion [40], while ten involved groups of health professionals and/or service staff, either a steering committee formed by local health services [38, 43, 44] or the “health centre” [27, 30, 41, 45–48]. A further two cases involved research and development units established by local health professionals or a local health service [31, 49]. Only four cases involved the participation of existing community-representative structures, including one where there was a health department within the local (county) government [29]. In eight cases, the innovation project itself established a new (and usually temporary) representative group whose structure was generally not well described.

### Roles and Phases

There was evidence of local actors in all of the roles in our framework. The most common role was as researcher/developer (28 cases) and many of the articles reviewed (*n =* 18) featured local actors as co-authors. Next most common (*n =* 25) was the stimulator-initiator role, and the similar role of facilitator (*n =* 20) was also common. Local ownership (*n =* 18) was apparent in more than half the articles, while only 13 referred to local roles that could be interpreted as relating to translation or maintenance (sustainability) of the initiative.

Every paper had information about local action in the phases of exploration and preparation (Table 6). There were only six cases where local actors (LAs) were not actively involved in initiating the project. Local actors were active participants in the preparation phase in all but four cases. Those four cases involved local health professionals collecting data to a prescribed template and having no (declared) role in data analysis or interpretation.

**TABLE 6:** Classification of innovation process phases (Sweden, Australia. 2022).

Phase	Examples
Exploration	LAs initiate the projectact (*n =* 13)
	LAs actively approve the project and agree to leadership role (*n =* 13)
	Project initiated independently of LAs (*n =* 6)
Preparation	LAs as researchers/co-researchers (recognized e.g., authors) (*n =* 18)
	LAs as active participants (collecting data, analysing, etc.) without formal recognition (*n =* 10)
	LAs as passive participants (*n =* 4)
Implementation	LA takes full responsibility (*n =* 6)
	Project partners continue to contribute (*n =* 5)
	New external partners (*n =* 2)
	Insufficient information (*n =* 19)
Sustainment	LAs evaluate (*n =* 7)
	New external evaluators (*n =* 6)
	Project partners evaluate (*n =* 1)
	No formal evaluation/insufficient information (*n =* 18)

It was much less common to have information about LALA at the “back end” of the innovation process. The reported research typically covered development activities (pilot testing, mostly), with no “re-visiting” of cases at later points in time. However, implementation was discussed in 13 articles, with local actors taking full responsibility for long term commercialisation of the initiative in six cases. Project partners (universities in all cases) continued to be involved in five cases [31, 32, 36, 49, 54], while in two cases [50, 51] new external partners were engaged to contribute to the implementation process. In terms of sustainment, there were six articles based on evaluations of past projects by researchers contracted for that task after the project had been commercialised, and one further case [51] where researchers involved in the development also conducted an evaluation.

### Impact of Local Actors and Local Actions on Innovation Processes and Outcomes

As expected, given that most articles only reported research to the end of the preparation phase, there was very little evidence of the impact of LALA on the outcomes of innovation projects. Many impacts are difficult to quantify, and difficult to attribute specifically to LALA [47]. Ka’opua et al. (2011) claimed that their process of enrolling women in a breast screening program using church leaders and church services led to increased screening rates, but we do not know if these persisted after the initial trial period [52]. Connor (2009) attributed a change in regional health funding policy (from project specific to “pooled funding”) to local action, but few details were provided [53].

The review may have identified impacts of LALA on intermediate processes. Four articles [29, 32, 40, 49] described how local actors were successful in attracting project extension or implementation funding. Three studies described a process of handing control-ownership over to local actors at the end of the project and suggested that it was only local actors who were in a position to provide for the project’s sustainment [36, 43, 50].

There was also some evidence of impacts of LALA on the nature of the projects. In Nykiforuk et al. (2018), local actors showed the researchers how an urban based walking program could be adapted to rural conditions [54]. In two studies it was local actors who were able to redirect health funding to support the project [35, 37]. In four cases [30, 46, 52, 53], the attachment of a local actor (usually a health professional) to a university research centre was seen as giving credibility to projects that had struggled for support.

There were four notable examples of LALA actively influencing the research and development (preparation) process. In Singer et al. (2015), local agitation led to a complete re-design of the trial of an Aboriginal mental health initiative in Australia [55]. In Andersen and Jansen (2012), local input changed what was intended to be a complex technology-enabled telepsychiatry model into a simple telephone-based initiative [50]. Stewart and Conway’s (2000) plan to engage communities in design and trial of a drink driving prevention program had to be changed when those actors were reluctant to commit as much time as the researchers wanted [56]. In Trout et al. (2018), the research protocol was changed significantly by the input of a local reference group which had concerns about cultural appropriateness [37].

## Discussion

This review set out to examine the role of local actors and local action (LALA) in the processes of health service innovation in small rural settings. A variety of types of innovation projects in these settings was discovered through the review, with a focus on public health, clinical interventions, and service models. Perhaps surprising was the lack of articles about eHealth innovation, which might reflect a more centralized process of eHealth diffusion [19]. While the review specifically sought articles which used “innovation” as a term, there were limited theoretical or conceptual links to innovation science. In particular, LALA was not conceptualized as a component of a “rural health innovation model” [5].

In this review, 20 of the 32 articles had health professionals or staff as local actors, suggesting a need to better understand how people manage their dual roles of health service and community representatives in innovation projects. Of interest here is the presence of two locally based and locally led health Research and Development (R&D) units (with another project [32] aiming at establishing a group). Hodge et al. (2016) have proposed the forming of such entities as a mechanism to manage local engagement with distant universities and health departments [22].

Where local actors were not health professionals, it was more common (8 out of 12 cases) to form new “representative” groups than to access existing structures. This might be a necessary strategy to encourage the representation of different parts of the community, but it also brings the risk of volunteer fatigue with individuals asked to engage with multiple projects through multiple structures. It can also de-legitimize existing structures or raise barriers to innovation adoption by marginalizing important groups. More formal and long-lasting governance structures would likely also have greater capacity to secure funding, re-allocate existing resources and advocate within the health system for embedding a new initiative.

The relatively high level of local ownership (Tables 2–4) is reassuring, particularly as one might expect “fully local” initiatives to be somewhat hidden from an academic literature more concerned with university-owned or led projects.

Notwithstanding the above comments on sustainment, it was difficult to quantify the impacts of LALA on innovation processes. The challenges of drawing links between LALA and health outcomes are similar to those described in the previous reviews of community participation, but in this review, there were clear LALA impacts on the design and implementation of the innovation project. Interestingly, on several occasions the impact of LALA was to “downsize” the project [45, 51, 54, 55], perhaps reflecting a greater ability of local actors to recognize the limits to change and to unwittingly adopt “anti-fragile” approaches to innovation. Those limits could be around what technologies could be sustained [50], what levels of community participation could be sustained [56], or what might be culturally appropriate [38, 55].

The review had some limitations that need to be acknowledged. The literature search was limited to high-resource countries and to articles which self-identified “innovations.” This perhaps biased towards North America where that language may be more common (although two of the three reviews of rural health innovation originated in Australia). Nevertheless, we saw evidence of LALA in innovation across a number of countries, suggesting that LALA can be achieved under a variety of health system/health policy settings. We also acknowledge that important lessons would be learned from LALA and innovation in lower-resource settings [2], and such a review would be a useful companion for this piece.

This review was also limited to small rural settings. The literature asserts that “standard” service models are unlikely to be sustainable in small rural contexts, heightening the need for innovation. However, the circumstances of each individual service are likely to be different in terms of threats to sustainability, meaning that local knowledge is critical to effective innovation. The need for innovation may be lower in urban contexts, and the assumed access to knowledge systems (universities, government agencies) higher. Exploring LALA in urban (or large rural) settings in contrast to small rural settings would be an interesting avenue for further research.

Again following Preston et al. [2], we recognize that the absence of discussion about LALA does not imply an absence of LALA, so what we are seeing here is the ways in which LALA has been documented, and this is likely to reflect only a small portion of what is done, particularly at the local ownership end of the continuum, where local actors (Patey et al. (2019) [31] as an example of a local R&D unit) may not see the need to describe their own roles.

We assumed that local health professionals were also local community members. We could have analyzed the extent that local health professionals were local community members (e.g., through length of residence, family connections, community participation, if authors self-identified as being members of the community, etc.). However, there was a lack of discussion of these dual roles (and reflexivity of authors). (Wright 2004 [46] gives an example of where there is discussion on these roles). The tension of these dual roles has been identified in the literature as problematic—but it is also problematic to draw a line between roles as health professionals and community members, particularly in small rural settings [18].

The review has contributed to understanding the potential for health services innovation in small rural settings, at least in high resource countries. Small rural communities, while lacking the depth of services of larger places, and while distant from the actors normally charged with driving innovation, can and do have the capacity to investigate, lead, own, and sustain innovation projects. And they might do so with more realistic understandings of what is possible in the local context. Promotion and facilitation of structures which can further increase LALA capacity (such as locally based R&D units) should be considered as part of rural health policy. Processes which facilitate the engagement of local actors with university-based researchers (including faculty appointments and industry-led research programs) should also be promoted. While there is much more research to be done into how to make LALA an effective contributor to innovative and sustainable models of health service delivery, there is evidence in the literature to this point to suggest that there is substantial scope for health service innovation in small rural settings that could be realized through engagement of local actors in local action.

## Abbreviations

LALA: local actors and local actions.
