# Supplementary material for: Innovation in Rural Health Services Requires Local Actors and Local Action
Source: Public Health Rev. 2022 Sep 14;43:1604921. doi: 10.3389/phrs.2022.1604921 (PMC9516414; doi:10.3389/phrs.2022.1604921)
Supplement: Supplementary file 1 [file DataSheet2.pdf]

Supplementary File 2: Summary of 32 Included Papers (Sweden, Australia. 2022)

**Paper**	**Small rural context**	***Type of innovation***<br>**Intervention**	***Type of Local Actors***<br>(*reference to other local participants)<br>**Local role**
Aadalen 1998 [33]	Arlington, Minnesota, USA (2233 residents)<br>Waseca, Minnestoa USA (9400)<br>Unnamed 3rd site of similar size<br>And urban partners – 60-100 miles away	*User/ patient engagement*<br>The main outcome was the implementation of a new nursing role. This paper reports on stage I (identifying the issues and proposing an intervention) and Stage II (the intervention).	*Health Professionals*<br>Nurse leaders<br>A project team featured nurses from each site. The team collectively analysed data about current state of play. The project team collectively identified potential strategies for improvement.).
Andersen & Jansen 2012 [50]	Finnmark county, Norway. So 'small rural' that it doesn't exist anymore (merged with Troms in 2020). Largest towns about 5000.	*eHealth/ technology*<br>Telepsychiatry model	*Informal community group*<br>Service leaders<br>"user-driven, bottom-up development process" (50p1).
Baloh et al 2018 [45]	8 'critical care' hospitals – 25 beds, Iowa, USA. These are typically more than 50km from the nearest larger hospital, and service catchments of 5-10 000 residents.	*Governance/ service model*<br>"Team huddles" – short briefing sessions at the start of a shift	*Health professionals*<br>The health center.<br>The hospitals had volunteered to implement team huddles, and this project simply assessed the effectiveness of their implementation process. So, the researchers were 'at a distance' from the innovation which was locally driven (and therefore different in each place).
Connor et al 2009 [53]	Reporoa / Kaingaroa, New Zealand. Town population 2000, service pop 3500.	*Governance/ service model*<br>Development of a "rural nursing service model"(49p4)	*Formal community group*<br>Health service governance committee<br>The senior nurse is one of the co-authors. The health service applied for 'innovation' funding from the Ministry of Health. So, it

Paper	Small rural context	*Type of innovation*<br>**Intervention**	*Type of Local Actors*<br>(*reference to other local participants)<br>**Local role**
			appears that it was a fully locally driven project.
Garney et al 2016 [41]	Leon County, Texas USA. 16 000 residents in total. Largest town <2000.	*eHealth/ technology*<br>Telepsychology initiative (counselling) – student-based service delivery model	*Health professionals**<br>The health centre<br>The County 'partnered' with Texas A&M, but we don't know who instigated that or the terms of partnership. Leon Health Resource Centre provided the space and client administration. The local service essentially ran the service, with the student counsellors as practitioners.
Greene et al 2020 [60]	Papago Indian Reservation, Arizona USA – Largest town (Gila) 10 000.	*eHealth/ technology*<br>Mobile health unit	*Formal community group*<br>Indian Health Service collaboration with NASA Lockheed Missiles and Space Company, members of the Papago (now Tohono O'odham) Tribe<br>"Role of the reservation as a site for knowledge production" (60 p447)
Halliday & Asthana 2005 [39]	Locations in West Cornwall, UK. "typically large villages with a population of 2000-6000"(40 p183).	*Prevention/ public health*<br>Three case studies – 1) setting up a new public health collaborative; 2) increasing public health role of visiting health practitioners, 3) a model for "family support, teenage pregnancy and parenting" (40 p185)	*Formal community group*<br>It was the local actors who wrote the grant applications, chose the projects, designed the interventions.
Haswell-Elkins et al 2009 [32]	Yarrabah and Hope Vale, Far North Queensland. Discrete Indigenous communities. <3000	*Knowledge/ capacity building*<br>Implementation of a Priority Driven Research (PDR) approach for a mental health research project. Normally we would exclude research projects, but	*Informal community group*<br>Depicts "the communities" as equal partners in identifying research needs, designing research projects, proposing responses to research findings etc. "The

**Paper**	**Small rural context**	***Type of innovation*** **Intervention**	***Type of Local Actors*** (*reference to other local participants) **Local role**
	and c.1000 pop respectively.	the innovation here is the establishment of a partnership for research which was intended to go beyond this particular project.	project team included five University-based and three community-based Aboriginal researchers/ service managers." (32[pS57])
Hatashita & Anderson 2004 [58]	Hanazono village, Japan (pop 600). In a relatively sparsely populated mountain tourism area. Kuchan (15 000 people) is 30km away. One hour to the nearest health service	*Governance/ service model* Employing a public health nurse.	*Informal community group* The paper states that "The village employed its first public health nurse in 1993" (58[p103]). It is not clear what structure within the village allowed that, however the nurse engaged in innovation (engaging in research projects, appointing community liaisons etc). Given that the nurse is now a local, we can see this as local action, and it is distinct because we know the nurse was in the 'small rural' and we have hints through the liaison persons, etc.
Horne et al 2013 [29]	Klickitat County, Washington State, USA. 20 000 people across c. 18 towns and villages. Largest 3 500.	*Prevention/ public health* A model (ACHIEVE) for preventing chronic disease (esp. related to obesity)	*Local government* The project was led by the Klickitat County Health Department. They won competitive funding and used a community-engagement model to design and implement the initiative.
Imboden & Fehr 2018 [49]	Southern Illinois, USA. Approx. 200 child ADHD patients in the region. 2-hour drive to pediatric care facility.	*Clinical intervention* ADHD management strategy	*Health sector research and development unit* 'Local practitioner' with university appointment The local nurse practitioner made contact with the university and was ultimately a co-researcher.

**Paper**	**Small rural context**	***Type of innovation*** **Intervention**	***Type of Local Actors*** (*reference to other local participants) **Local role**
Jacklin et al 2020 [59]	Manitoulin Island, Ontario, Canada. Population 14000 across c. 15 towns and villages.	*Clinical intervention* Dementia testing procedure for Indigenous communities.	*Informal community group* Four community participant groups were established to guide the adaptation of the testing procedure to this setting. Community groups provided feedback on the procedure.
Kao'pua et al 2011 [52]	Wai'anae Hawaii – population 13 000	*Clinical Intervention* Breast cancer screening intervention	*Formal community group* Co-designed with local community (elders and church leaders) to make the delivery culturally appropriate. No real evidence of how the delivery itself was different, but the environment in which the delivery occurred was novel and locally determined.
Kunz et al 2017 [47]	Santa Cruz County, Arizona USA. Pop 50000 across 10 or so towns and villages. Largest town 25 000.	*Prevention/ public health* Diabetes prevention and management program	*Health professionals** The health centre Lists local actors and their roles. Operated as a formal consortium.
Maar et al 2009 [57]	Manitoulin Island, Ontario Canada	*Clinical intervention* Mental health initiatives (several)	*Informal community group* Positions the local mental health team as the innovators, and this study is about describing the history of innovation.
Maurana et al 2000 [34]	Marion area of Wisconsin, USA. Population c. 2000.	*Prevention/ public health* Using medical students for public health activities	*Formal community group* Positions 'the community' as co-developers in terms of goal setting, developing programs.
McConigley et al 2011 [44]	Great Southern Region of Western Australia. Total population	*Clinical intervention* Western Australia Cancer Network – series of projects for cancer care	*Health professionals** Steering committee formed by local health services

Paper	Small rural context	*Type of innovation*<br>**Intervention**	*Type of Local Actors*<br>(*reference to other local participants)<br>**Local role**
	50 000, no town larger than Albany 30 000. The project focuses on the smaller centres.		Project led by a local steering committee.
Moore et al 2016 [38]	Missouri, USA – Lafayette County 33 000 largest town 4000<br>Cass County – Population 100 000, largest town 10 000, but part of the Kansas City Metro Area.<br>Allen County, Kansas – 13 000 pop, largest town 6 000	*Knowledge/capacity building*<br>Initiative to support development of rural health initiatives	*Health professionals**<br>Steering committee formed by local health services<br>The paper focuses on how local input changed the planned initiative of local leadership groups
Morgan et al 2019 [43]	Sun Country Health Region, Saskatchewan, Canada. 60 000 pop largest town 11 000.	*Clinical intervention*<br>Dementia management intervention	*Health professionals**<br>Steering committee formed by local health services<br>A memory clinic model was co-designed via local focus groups
Neufeld & Case 2013 [48]	Bowen Health Center, across 20 sites in Indiana, USA – some are small rural	*eHealth/ technology*<br>Walk-in telemental health clinics	*Health professionals*<br>The health centre<br>The walk-in centers are in the smaller sites that implemented the services. The Center invented everything.
Nykiforuk et al 2018 [54]	Bonnyville, Alberta Canada. Population 6000	*Prevention/ public health*<br>Program to promote walking	*Informal community group*<br>"After seeing their data, Bonnyville community partners prioritized the

Paper	Small rural context	*Type of innovation* **Intervention**	*Type of Local Actors* (*reference to other local participants) **Local role**
			development of a local, evidence-informed 'pedestrian-friendly' walking map."(54[p216]) and set priority user group targets. University helped with data collection
Patey et al 2019 [31]	Carbonear, Newfoundland and Labrador, Canada. Population 5000	*Governance/ service model* New patient flow management model	*Health sector research and development unit* Carbonear Institute for Rural Research and Innovation by the Sea All done within the local hospital, in partnership with a university at urban centre.
Pesut et al 2017 [51]	Castlegar, British Columbia, Canada. Population 8000 plus a small catchment and a similar sized (unnamed) community nearby	*Governance/ service model* Nurse-led navigation to provide early palliative care	*Informal community group* A local innovation originating from the local college.
Rivara et al 1983 [35]	Town of Hazard in Kentucky, USA. Population c. 5000	*Prevention/ public health* Home visit program for newborns	*Health Professionals* Nurse leaders "…the Frontier Nursing Services (FNS) had a long history of innovation and alternative delivery methods." [35 [p46]]
Schofield & Bourgeois 2010 [36]	North-eastern half of New Brunswick, Canada. "only a handful of towns or villages have a population exceeding 5000 residents."(p265)	*Knowledge/ capacity building* Medical education initiative	*Health professionals** Nurse leaders Local medical leaders with local university championed a local medical school for 30 years.

Paper	Small rural context	*Type of innovation*<br>**Intervention**	*Type of Local Actors*<br>(*reference to other local participants)<br>**Local role**
Shaikh et al 2015 [40]	Rural communities in California, USA. There is a map, but it is illegible. But the table summarising the clinical context suggests strongly small rural – maximum 3 physicians, some clinics staffed by nurse practitioners and physician assistants.	*Prevention/ public health*<br>Childhood obesity prevention initiative	*Health Professionals*<br>Clinical champions<br>Developed a multidiscipline community of practice.
Singer et al 2015 [55]	Ngayundi Health District, northern New South Wales, Australia. Bundjalung and Yaegl nations – which do include Tweed Heads, but is otherwise exclusively small rural.	*Prevention/ public health*<br>Aboriginal mental health initiative	*Informal community group*<br>Describes how local actors can be engaged in re-designing what was a 'top down' service.
St Pierre-Hansen et al 2010 [30]	Sioux Lookout, Ontario Canada. Population 5000	*Clinical intervention*<br>Palliative care model for First Nations communities	*Health professionals**<br>The health center<br>The local health service is credited with developing its own model of care, with "…patient surveys; an elders' council; and consultations with community members, community leaders, and a traditional healer" (30[p.42]).
Stewart & Conway 2000 [56]	Te Awamutu district, Waikato, New Zealand.	*Prevention/ public health*<br>Drink driving reduction program	*Formal community group*

Paper	Small rural context	*Type of innovation*<br>**Intervention**	*Type of Local Actors*<br>(*reference to other local participants)<br>**Local role**
	"several small towns of less than (sic) 10 000 population" (p143)		Local actors proposed ideas, approved of plans and had some management responsibilities
Trout et al 2018 [37]	11 villages in Alaska USA ranging from 90 to 1200 population.	*Prevention/ public health*<br>Indigenous youth suicide prevention	*Health professionals**<br>The Health Center<br>Health service is a "tribally-governed health and social services organization" (37[p397])
Ulrich et al 2019 [42]	Cane Creek North Carolina USA. Pop < 1000	*Governance/ service model*<br>Pharmacist-physician co-visit model	*Health Professionals*<br>Pharmacist<br>Project was run by the local clinic.
Wright 2009 [46]	USA<br>Clairfield, Tennessee (pop 1100)<br>Franklin, Louisiana (8000)<br>Rio Arriba County New Mexico (40 000 – largest town 10 000)<br>Brownsville, California (1000)	*Governance/ service model*<br><br>Individual rural primary care models in 4 locations	*Health Professionals**<br>The health center<br>Explores why these initiatives succeeded when others failed, including aspects of local action.
