# Supplementary figures and images for: Innovation in Rural Health Services Requires Local Actors and Local Action
Source: Public Health Rev. 2022 Sep 14;43:1604921. doi: 10.3389/phrs.2022.1604921 (PMC9516414; doi:10.3389/phrs.2022.1604921)

Supplementary File 1: Review process (Sweden. 2022)


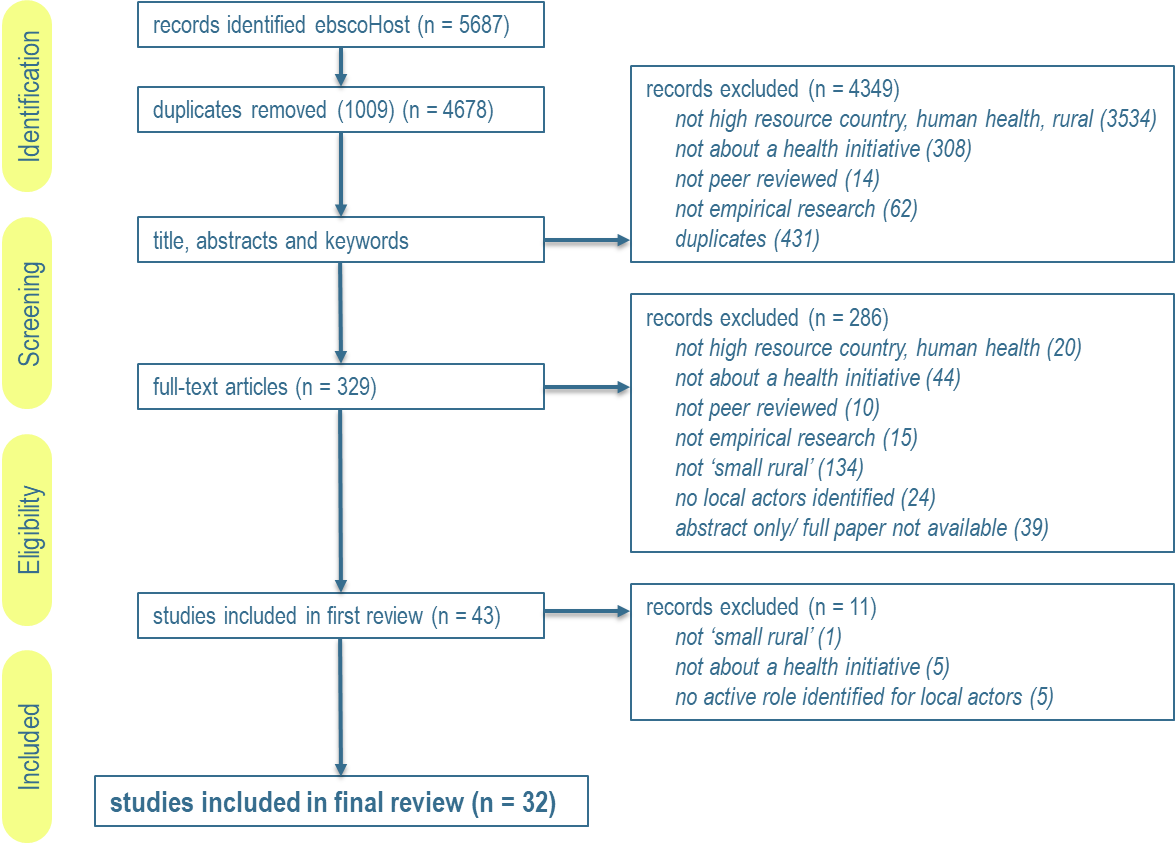

Supplement: Supplementary file 2 [file DataSheet1.docx]
